# Characteristics of Children with Type 1 Diabetes and Persistent Suboptimal Glycemic Control

**DOI:** 10.4274/Jcrpe.663

**Published:** 2012-06-09

**Authors:** Hyuntae Kim, Angelo Elmi, Celia L. Henderson, Fran R. Cogen, Paul B. Kaplowitz

**Affiliations:** 1 George Washington University School of Public Health and Health Services, Department of Epidemiology and Biostatistics, Washington, D.C., USA; 2 Children's National Medical Center, Department of Endocrinology and Diabetes, Washington, D.C., USA; +202 476 3808 pkaplowi@cnmc.org

**Keywords:** type 1 diabetes mellitus, glycosylated hemoglobin, glycemic control, children

## Abstract

**Objective:** This study aims to determine the relationship between the duration of persistent poor glycemic control in type 1 diabetes mellitus (T1DM) children and the likelihood of subsequent improvement.

**Methods:** A retrospective cohort study was conducted on T1DM patients aged 6-18 years, followed for at least six visits at Children’s National Medical Center (Washington, DC) with at least one hemoglobin A1c (HbA1c) ≥10% after the first year since the initial visit (n=151). Medical records of patients with subsequently improved glycemic control were reviewed (n=39).

**Results:** Patients aged 12-18 years, females, and Medicaid patients were twice as likely to be in persistently poor control as patients aged 6-11 years, males, and privately insured patients, respectively. Each additional visit with HbA1c ≥10% and one percentage point increase in the mean HbA1c reduced the likelihood of subsequent improvement by 20% and 50%, respectively. Of the 39 patients with improved control, only 5 (13%) sustained their improvement for ≥2 years. Multiple contributing factors for improved control were identified, but no one factor explained improved control in >25% of patients.

**Conclusion** This study suggests that the longer the duration of poor control, the more difficult it is to reverse the underlying factors of poor diabetes management. Strategies to improve regular clinic attendance along with reinforcement of changes which resulted in improved control are critical. Adolescents, females, and Medicaid patients in particular should be targeted for sustained intervention.

**Conflict of interest:**None declared.

## INTRODUCTION

Successful management of type 1 diabetes mellitus (T1DM) requires sustained collaboration involving the patient, family, caregivers, and the multidisciplinary diabetes team. Near normalization of blood glucose often fails even in patients and families who consistently perform self-care skills including the prescribed insulin regimen and diet (1). Children and adults with well-managed diabetes typically have hemoglobin A1c (HbA1c) levels in the range of 6-8%, approximately equivalent blood glucose levels in the range of 120-180 mg/dL. On the other hand, patients with poor diabetes management have a much higher HbA1c level, typically ≥10%. These high values are estimated to reflect a 2-3 month blood sugar average of ≥240 mg/dL.

Children with T1DM present unique challenges due to problems in diabetes self-care responsibilities which arise between the ages of 10 and 15, often resulting in deterioration of glycemic control. Factors which have been found to be predictive of HbA1c levels include age (2) and gender (3), regularity of clinic attendance (4), frequency of blood glucose monitoring (5,6), number of insulin injections (7), and the duration of diabetes (8) Diabetes self-management occurs within the home environment and is consequently influenced by social and economic stressors. Family factors (9), psychological characteristics (10), and economic issues (11) all may influence glycemic control.

These identified factors, however, have not been well studied in the subgroup of children with T1DM who have persistent difficulty with their diabetes management. Noting that this particular group of patients is at higher risk for long-term complications (7) and therefore likely to be responsible for a significant portion of medical costs associated with T1DM, it is important to study this population in more depth. The objectives of this study were to characterize children with T1DM and persistent poor control, to determine the relationship between the duration of poor control and the likelihood of subsequent improvement, and to identify factors associated with improved control.

## METHODS

**Study Design**

We conducted a retrospective cohort study using the Clinipro® (NuMedics, Tigard, OR, USA) database, which contained clinical data of all patients who had attended the diabetes clinic at Children’s National Medical Center (CNMC) in Washington, D.C. from Jan 1st, 2002 to Jan 1st, 2011. Patients with T1DM were eligible for inclusion if their average age for the duration of follow-up was 6-18 years , if they had been followed for at least 6 clinic visits and had at least one HbA1c ≥10% result after the first year since their first visit to CNMC. The last criterion was used to exclude patients with a single HbA1c value ≥10% that was obtained on a sample taken at the time of diagnosis and therefore could not reflect poor diabetes management. In our study, a patient’s glycemic control was considered to have significantly improved if his/her HbA1c level was reduced to below 9% or by at least 3% below the peak after two or more consecutive visits with HbA1c levels of ≥10% (i.e., persistent poor control). A patient’s glycemic improvement was considered sustained if the patient’s HbA1c level was continuously maintained below 10% for two years subsequent to the decrease or not sustained if the HbA1c level documented at the time of improvement increased by ≥2% at the next visit or by ≥3% at the second visit following the nadir.

**Data Sources**

Patients were generally seen every 3-4 months; however, there were much longer time gaps between clinic visits for some patients due to missed appointments. All data were entered by trained personnel into CliniPro®. HbA1c levels were determined using the DCA 2000®+ HbA1c System (Bayer, Tarrytown, NY, USA) with results available during the visit. The highest measurable HbA1c reading with the DCA 2000®+ HbA1c System was 14%. The medical charts of patients with improved control were reviewed to identify factors that have led to improvement, including demographic and clinical characteristics as well as modifications that patients claimed to have made prior to the visit with documented improved control (e.g., increased parental supervision).

**Statistical Analysis**

The frequency distribution and the mean and standard deviation of the demographic and clinical characteristics were examined for all included patients and for patients with persistent poor control separately. Additionally, after review of selected charts, the frequency of each type of modification was tallied for patients with persistent poor control and subsequent improvement. Chi-squared tests or Fisher’s exact tests were used to test the difference in proportion within each nominal independent variable by the dependent variable of interest, whether or not improved control had occurred after an extended period of poor diabetes management. Two sample t-tests or one-way analysis of variance (ANOVA) were used to test the difference in mean of each continuous independent variable by categorical independent variables and, separately, by our principal dependent variable. The bivariable relationships between continuous independent variables using Pearson’s correlation analysis were examined for multicollinearity. A variable with the highest correlation only was entered into the multivariable logistic regression model. Multivariable logistic regression analysis with the demographic and clinical characteristics, along with their 2nd order interaction terms, controlling for age, gender, race, and insurance, was performed to examine the relationships of our variables with the odds of subsequent improvement in glycemic control. Model selection was based on the stepwise method. All statistical analyses were performed using SAS version 9.1.3 (SAS Institute Inc., Cary, NC, USA).

**Human Subjects Protection Issues**

This study was approved by the Institutional Review Board at CNMC. The only type of protected health information used in our study was a medical record identification number, which was needed to cross-match select patients to their medical charts. 

## RESULTS

**Characteristics of Included Patients**

The majority of the patients followed at CNMC for T1DM have been able to maintain fair to good glycemic control over time and were therefore not included in our analysis. There was, however, a sizable subset of 151 patients who met the inclusion criteria (Table 1). The mean average age (± SD) of the included patients was 12.7±2.7 years, 65% being between 12-18 years, and the rest between 6-11 years old (χ2=13.41; p=0.0003). There were 52% African-Americans, 23% Caucasians, and 13% Hispanics in the included group, while the entire database (n=2.312) had 54% Caucasians, 31% African-Americans, and 7% Hispanics. Both genders and insurance groups (Medicaid vs. private insurance) were represented nearly equally (p>0.05 for all). The majority of the included patients (67%) attended the clinic 3-4 times/yr, whereas the remainder was seen less than 3 times/yr (χ2=17.23; p<0.0001). The mean HbA1c level (±SD) of African-Americans (11.2±1.3%) did not differ from that of Hispanics (10.9±1.1%), but was significantly higher than that of Caucasians (10.1±1.3%) (F=6.64; df=3; p=0.0003).

**Characteristics of Patients in Persistently Poor Glycemic Control**

Of the included patients, 104 (69%) were found to have ≥2 consecutive visits with HbA1c levels of ≥10% (i.e., persistent poor control) (Table 2). The Hispanic group had the highest proportion of Medicaid patients (87%), followed by African-American (53%) and Caucasian (32%) (χ2=12.49; df=3; p=0.006). The racial group with the most regular follow-up was Hispanic (87%) whereas only 58% of Caucasians and 55% of African-Americans were seen ≥3 times/yr (χ2=8.36; df=3; p=0.038). The mean HbA1c level (±SD) of patients with ≤2 visits per year (11.6±1.1%) was slightly but significantly higher compared to those with ≥3 visits per year (11.0±1.3%) (F=5.62; p=0.020). Patients between 12 and 18 years of age were twice as likely to be in persistently poor control [odds ratio (OR)=2.07 (1.02, 4.22); χ2=4.11; p=0.043] and to be higher in HbA1c level (11.0±1.3% vs. 10.3±1.3%; t=-3.16; p=0.002) as those between 6 and 11 years of age. Compared to boys, girls were twice as likely to be in persistently poor control [OR=2.11 (1.04, 4.27); χ2=4.40; p=0.036] and to have a higher mean HbA1c level (11.0±1.4% vs. 10.5±1.3%; t=2.23; p=0.028). Medicaid patients were also twice as likely to be in persistently poor control as patients with private insurance [OR=1.81 (1.13, 2.90); χ2=7.54; p=0.006]. No significant correlation was found among the variables that we examined (p>0.05).

**Characteristics of Patients in Persistently Poor Glycemic Control with Subsequent Improvement**

Of the 104 patients in persistently poor control, 39 (38%) subsequently had a significant improvement in their HbA1c levels (Table 3). The mean HbA1c level (± SD) of the patients who had a significant decrease in HbA1c (10.7±1.2%) was significantly lower than that of patients whose control did not improve (11.5±1.2%) (t=3.32; p=0.001). The mean duration (±SD) of persistent poor control for the patients with subsequent improved control (3.9±2.2 visits) was significantly shorter compared to those without improvement (5.9±3.4 visits) (t=3.63; p=0.0004). Patients with only one visit with an HbA1c level ≥10% were two times more likely to have subsequent improved control than those with two or more consecutive visits with HbA1c levels of ≥10% (OR=2.04 (1.52, 2.74); χ2=19.79; p<0.0001).Sixty-four percent of patients with two consecutive visits with HbA1c levels ≥10%, 37% of those with three such visits, and 29% of those with ≥4 such visits subsequently improved (Z=2.832; p=0.005). Our patients with only two consecutive visits with HbA1c levels ≥10% were over four times more likely to have improved compared to those with ≥4 such visits [OR=4.38 (1.40, 14.06); χ2=8.37; df=2; p=0.004]. There was no significant difference in age, gender, race, insurance, and the average number of visits per year between the two outcome groups (p>0.05 for all). A significant positive correlation was observed between the duration of persistent poor control and age at the time of improvement (R2=18.98; p=0.006). Patients aged 12-18 years (4.2±2.3 visits) had a longer period of persistent poor control than patients aged 6-11 years (2.4±0.8 visits) (t= - 3.41; p=0.002).

Five (13%) patients with persistent poor control were found to have sustained their improvement for ≥2 years, whereas 21 (54%) patients showed worsening of control within the next two years after improved control (Table 3). Neither worsening nor sustained improvement could be documented among the remaining patients (33%) because the database only had records of visits for one year or less after the visit at which improved control was noted. Medical chart reviews revealed that increased parental supervision (23%), improved overall adherence (23%), self-improved diet (23%), nutritionist visit (18%), and fewer insulin injections per day (18%) were among the most common modifications in those patients whose control improved. In very few cases (≤5%), did we find that a significant increase in physical activity or counseling visit was documented at the visit when the decreased HbA1c level was observed. A single modification was given as a contributing factor of improved control for eleven (28%) patients, whereas 18 (46%) reported to have made more than one modifications prior to the visits with documented improved control. For the remaining 10 (26%) patients, no modification of the diabetes regimen was documented in the medical record.

**Predictors of Significant Improvement in Glycemic Control**

The final multiple logistic regression controlling for age, gender, race and insurance showed that the duration of persistent poor control and the mean HbA1c level were significant in predicting whether a persistently poorly controlled patient would subsequently improve in glycemic control (Table 4). Each additional visit with HbA1c ≥10% and every one percentage point increase in the mean HbA1c level reduced the likelihood of subsequent improved control by 20% and 50%, respectively. 

## DISCUSSION

Previous studies support our conclusions that older children tend to have more difficulty of diabetes control than younger children (12,13,14). In addition, we were able to quantify the observation that, once glycemic control had deteriorated, this population was very likely to remain in poor control for extended periods of time. The reason for both observations appears to be similar. According to previous studies, the changing hormonal milieu of adolescents with T1DM, combined with decreased adherence, places them at risk for higher HbA1c levels (12,13). The situation is often exacerbated by the fact that adolescents are expected to assume increasing responsibility for their own diabetes care with subsequent decreased supervision by caregivers for blood glucose monitoring, insulin administration and dose adjustment (14). If such issues persist, patients may have persistent poor control, despite efforts by the diabetes team to suggest strategies to reverse the poor control at clinic visits.

Moreover, we found that young females were more likely to be in persistent poor control than young males. Decreased insulin sensitivity that occurs during puberty, particularly in females (12,15,16), and psychosocial factors, such as the level of adjustment to illness (17), may play an additional role in sustained poor diabetes management. Eating disorders are also more prevalent in young females with T1DM than in those without T1DM (18,19), and than in young males with T1DM (20). Eating disorders in young females with T1DM are associated with insulin omission (21,22,23), severe dietary indiscretion (23), pervasive noncompliance with medical treatment (23), and poor glycemic control (19,21,22,23).

In addition, our finding that more patients covered by Medicaid were in persistently poor control than those with private insurance likely reflects a wide array of social and financial challenges. Medicaid insurance has been related to an increased risk of severe hypoglycemia (24) and diabetic ketoacidosis (25). However, sociodemographic factors associated with Medicaid (e.g., low income, single parenting), rather than Medicaid itself, may be accountable for such challenges as T1DM care and management requires an extensive amount of both tangible and non-tangible resources, such as ready availability of insulin and diabetes supplies and parental supervision (11).We found that an increase in the duration of persistent poor control or in the mean HbA1c level significantly reduced the likelihood of subsequent improvement. This finding suggests that immediate attention should be given to identifying possible causes of declining diabetic control and to instituting changes in diabetes management to address them as soon as deterioration of control is identified. Because physicians in the United States typically see children with T1DM every three to four months and missed visits are especially common in patients with poor control, this finding reinforces the importance for the patients of regular diabetes clinic attendance. Jacobson and colleagues compared 9- to 16-year-old children who visited the diabetes clinic on a regular basis to those who had irregular follow-up. They found that irregular follow-up was associated with worse glycemic control in the first, second, and third year of the study (4). In our sample, the patients who were less frequently seen (≤2 visits/yr) had significantly higher mean HbA1c levels compared to those with regular clinic attendance (≥3 visits/yr).

Through our chart reviews of patients whose glycemic control improved significantly after persistent poor control, factors that were most often associated with improved control and sustained improvement were identified. Although clear requests for parents to supervise the administration of insulin is one of our most frequent interventions, we found that in only 23% of our sample was increased supervision felt to be a factor in the child’s improved control (since we did not review charts where poor control did not improve, we cannot estimate how often this strategy was successful). Only seven of our 39 patients with improved control received nutritional counseling, even though many more had been referred to a dietitian. It is of interest that only two of our nine patients who reported improved diet had actually been seen by a dietitian. This suggests that when patients are motivated enough to take better care of their diabetes, they may already have adequate knowledge about healthier eating patterns to make relevant changes (e.g., giving up sugar-sweetened beverages and sweet snack foods) on their own. There were seven cases (18%) where a decrease in the number of injections (typically from 3-4 injections/day to 2 injections/day) was followed by improved control, suggesting that, in some cases, reducing the potential for missed doses is beneficial. In contrast, no cases were identified where a significant reduction in HbA1c level occurred after the number of injections/day was increased, as when patients were switched from a conventional split and mixed insulin regimen to a basal/bolus regimen.Because our study utilized retrospectively gathered data spanning approximately nine years, it is subject to potential sources of error. Even though we attempted to limit measurement error by using a standardized data collection tool, there may have been inaccuracies in the original documentation in the medical records. For instance, changes in insulin regimen were well documented, but changes in parental supervision were not and therefore likely under-reported. This tendency to document one particular aspect more frequently than the other might have led to over-reporting, which likely produces differential information bias. Most of these errors, however, are non-differential with respect to improvement and regression in glycemic control and will most often cause a result to be biased toward the null hypothesis. This source of bias may have led us to miss potentially significant associations.

There are several limitations associated with our study design and methods as well. We cannot exclude the possibility that no relationship was observed because of our relatively small sample size of patients with documented substantial improvement in HbA1c level. In attempting to study the subgroup of patients in persistent poor control, we arbitrarily selected an HbA1c level of ≥10%, reflecting an average blood glucose level of approximately 240 mg/dL. HbA1c is a widely used measure of glycemic control, but it may imperfectly correlate in some patients with average blood glucose levels over the previous months (26). Thus, some patients with an HbA1c level of 9% may be in as poor control based on actual blood glucose levels as other patients with an HbA1c value of 11%. We also used an arbitrary definition of improved control (i.e., a reduction in HbA1c level to below 9% or by at least 3%). For example, a patient whose HbA1c level dropped from 14% to 10.5% was considered to have improved under our definition, even if the end result remained unsatisfactory. Also, our accuracy at determining the extent of improved control may have been limited by the fact that the highest HbA1c level which can be measured on the DCA 2000®+ HbA1c system is 14%. The relatively small number of patients who demonstrated improved glycemic control after persistent poor control reduces the ability to ascertain whether the findings concerning which modifications may have caused the sustained improvement are statistically significant. In addition, the duration of diabetes was excluded from all levels of analysis examining the likelihood of subsequent improvement in glycemic control. It was challenging to define the duration of diabetes for our patients because their period of follow-up was long and varied widely among subjects. Lastly, we did not conduct medical chart reviews on the large number of patients who did not show improvement in glycemic control, which would have allowed us to compare them to the patients with improvement in terms of each documented modification. However, it was not practical to review every clinic letter of the included patients.

Our study demonstrated that patients who had at least one visit with HbA1c ≥10% but were not in persistently poor control were more likely to have subsequently improved than those with persistent poor control. We also demonstrated that older age, gender, and Medicaid insurance were independently associated with the likelihood of persistent poor control. We established that the longer children were in persistently poor control or the higher HbA1c level they had on average, the less likely they would subsequently improve. In addition, we found that improvement in glycemic control was often not sustained for extended periods of time. Our findings confirmed the importance of regular clinic attendance (3,4,8). Strategies must be developed to improve accessibility to the clinic and to identify patients who frequently miss appointments. Moreover, additional time may need to be spent at visits during which improvement in glycemic control is documented, in order to reinforce the changes in diabetes management that were responsible for the observed improvement, which may increase the likelihood that such improvement will be sustained.

## Figures and Tables

**Table 1:**
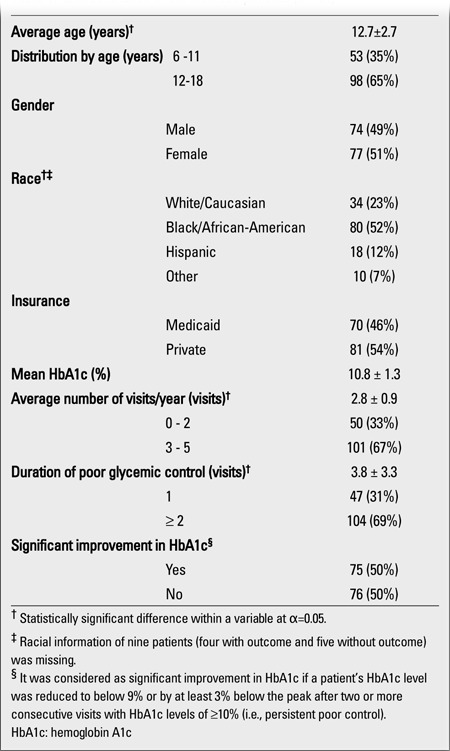
Characteristics of the included patients (n=151)

**Table 2:**
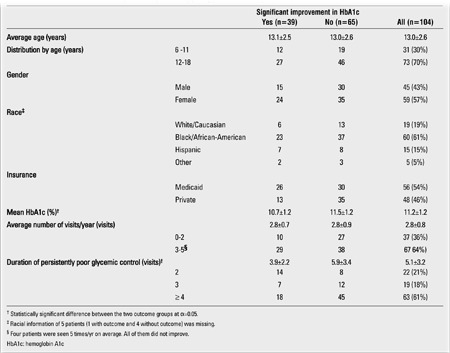
Characteristics of the included patients in persistently poor glycemic control by the outcome variable (n=104)

**Table 3:**
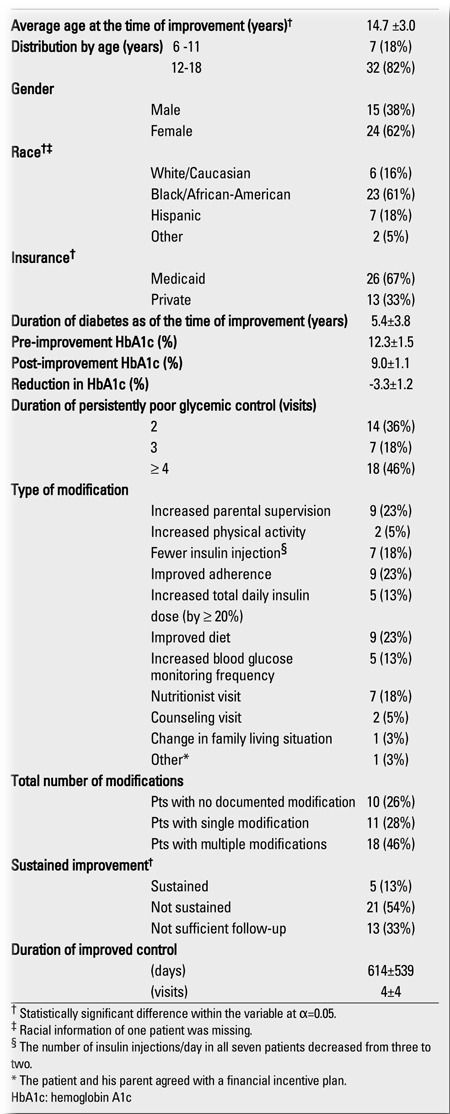
Characteristics of the included patients in persistently poorglycemic control who subsequently improved (n=39)

**Table 4:**
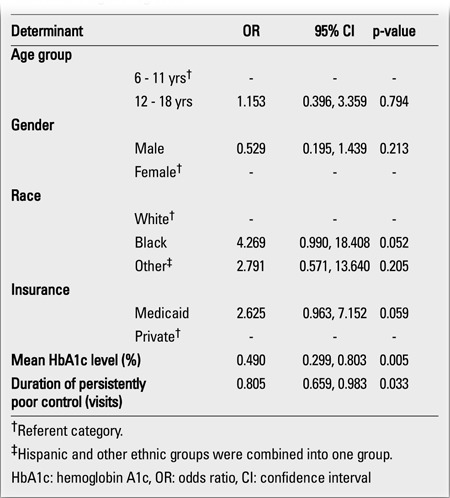
Determinants of the likelihood of subsequent improved control inchildren with type 1 diabetes and persistently poor glycemic control, usingmultivariable logistic regression

## References

[1] Mehta SN, Wolfsdorf JI (2010). Contemporary management of patients with type 1 diabetes. Endocrinol Metab Clin North Am.

[2] Daneman D, Wolfson DH, Becker DJ, Drash AL (1981). Factors affecting glycosylated hemoglobin values in children with insulin-dependent diabetes. J Pediatr.

[3] Mortensen HB, Robertson KJ, Aanstoot HJ, Danne T, Holl RW, Hougaard P, Atchison JA, Chiarelli F, Daneman D, Dinesen B, Dorchy H, Garandeau P, Greene S, Hoey H, Kaprio EA, Kocova M, Martul P, Matsuura N, Schoenle EJ, Sovik O, Swift PG, Tsou RM, Vanelli M, Aman J (1998). Insulin management and metabolic control of type 1diabetes mellitus in childhood and adolescence in 18 countries. Diabet Med.

[4] Jacobson AM, Hauser ST, Willett J, Wolfsdorf JI, Herman L (1997). Consequences of irregular versus continuous medical follow-up in children and adolescents with insulin-dependent diabetes mellitus. J Pediatr.

[5] Dorchy H, Roggemans MP, Willems D (1997). Glycated hemoglobin and related factors in diabetic children and adolescents under 18 years of age: a Belgian experience. Diabetes Care.

[6] Levine BS, Anderson BJ, Butler DA, Antisdel JE, Brackett J, Laffel LM (2001). Predictors of glycemic control and short-term adverse outcomes in youth with type1 diabetes. J Pediatr.

[7] The Diabetes Control and Complications Trial Research Group (1993). The effect of intensive treatment of diabetes on the development and progression of long-term complications in insulin-dependent diabetes mellitus. N Engl J Med.

[8] Rosilio M, Cotton JB, Wieliczko MC, Gendrault B, Carel JC, Couvaras O, Ser N, Gillet P, Soskin S, Garandeau P, Stuckens C, Le Luyer B, Jos J, Bony-Trifunovic H, Bertrand AM, Leturcq F, Lafuma A, Bougneres PF (1998). Factors associated with glycemic control: a cross-sectional nationwide study in 2,579 French children with type 1diabetes. Diabetes Care.

[9] Kaufman FR, Halvorson M, Carpenter S (1999). Association between diabetes control and visits to a multidisciplinary pediatric diabetes clinic. Pediatrics.

[10] Rovet JF, Ehrlich RM (1988). Effect of temperament on metabolic control in children with diabetes mellitus. Diabetes Care.

[11] Songer TJ, LaPorte R, Lave JR, Dorman JS, Becker DJ (1997). Health insurance and the financial impact of IDDM in families with a child with IDDM. Diabetes Care.

[12] Amiel SA, Sherwin RS, Simonson DC, Lauritano AA, Tamborlane WV (1986). Impaired insulin action in puberty: A contributing factor to poor glycemic control in adolescents with diabetes. N Engl J Med.

[13] Anderson B, Ho J, Brackett J, Finkelstein D, Laffel L (1997). Parental involvement in diabetes management tasks: relationships to blood glucose monitoring adherence and metabolic control in young adolescents with insulin-dependent diabetes mellitus. J Pediatr.

[14] Gordon CM, Mansfield MJ (1996). Changing needs of the patient with diabetes mellitus during the teenage years. Curr Opin Pediatr.

[15] Lane PH (2002). Diabetic kidney disease: impact of puberty.. Am J Physiol Renal Physiol.

[16] loch CA, Clemons P, Sperling MA (1987). Puberty decreases insulin sensitivity. J Pediatr.

[17] Grey M, Davidson M, Boland EA, Tamborlane WV (2001). Clinical and psychosocial factors associated with achievement of treatment goals in adolescents with diabetes mellitus. J Adolesc Health.

[18] Smith FM, Latchford GJ, Hall RM, Dickson RA (2008). Do chronic medical conditions increase the risk of eating disorder? A cross-sectional investigation of eating pathology in adolescent females with scoliosis and diabetes. J Adolesc Health.

[19] Affenito SG, Adams CH (2001). Are eating disorders more prevalent in females with type 1 diabetes mellitus when the impact of insulin omission is considered. Nutr Rev.

[20] Grylli V, Hafferl-Gattermayer A, Schober E, Karwautz A (2004). Prevalence and clinical manifestations of eating disorders in Austrian adolescents with type 1 diabetes. Wien Klin Wochenschr.

[21] Pollock-BarZiv SM, Davis C (2005). Personality factors and disordered eating in young women with type 1 diabetes mellitus. Psychosomatics.

[22] Garcia-Reyna NI, Gussinyer S, Raich RM, Gussinyer M, Tomas J, Carrascosa A (2004). Eating disorders in young adolescents with type 1 diabetes. Med Clin (Barc).

[23] Pollock M, Kovacs M, Charron-Prochownik D (1995). Eating disorders and maladaptive dietary/insulin management among youths with childhood-onset insulin-dependent diabetes mellitus. J Am Acad Child Adolesc Psychiatry.

[24] Allen C, LeCaire T, Palta M, Daniels K, Meredith M, D'Alessio DJ (2001). Wisconsin Diabetes Registry Project. Risk factors for frequent and severe hypoglycemia in type 1 diabetes. Diabetes Care.

[25] Mallare JT, Cordice CC, Ryan BA, Carey DE, Kreitzer PM, Frank GR (2003). Identifying risk factors for the development of diabetes ketoacidosis in new onset type 1 diabetes mellitus. Clin Pediatr (Phila).

[26] Cohen RM, Smith EP (2008). Frequency of HbA1c discordance in estimating blood glucose control. Curr Opin Clin Nutr Metab Care.

